# Sharp-Pointed Memories

**Published:** 1870-04

**Authors:** 


### SHARP-POINTED MEMORIES.

The mind can kill the body. Remorse for wrong-doing has laid many a victim in the grave. There are mental tortures more terrible than the gallows or the guillotine. Many a man walks the earth a living skeleton, because the fires of memory are eating him up, and for such things there is no cure this side of the grave. Prevention is the great wisdom, and youth the time for its application. The rules of conduct should be

First. Act deliberately.
Second. Do nothing for revenge.
Third. Perish rather than wrong another.

An old man writes, —

"A number of us school-boys were playing by the roadside one Saturday afternoon, when the stage-coach drove up to a neighboring tavern, and the passengers alighted. As usual, we gathered around to observe them. Among the number was an elderly man with a cane, who got out with much difficulty, and, when on the ground, he walked with the most curious contortions. His feet turned one way, his knees another, and his whole body looked as though the different limbs were independent of it and each other, and every one was making motions to suit itself. I unthinkingly shouted, 'Look at old Rattlebones!' while the poor man turned his head with an expression of pain which I can never forget.

"Just then, to my surprise and horror, my father came around the corner, and immediately stepping up to the stranger, shook his hands warmly, and assisted him to walk to our house, which was but a little way off. I could enjoy no more play that afternoon, and when tea-time came I would gladly have hid myself, for I knew that he would be in, and so tremblingly went into the sitting-room. To my great joy and relief, the stranger did not recognize me, but remarked pleasantly to my father as he introduced me: — 'Such a fine boy was surely worth saving.'

"How the words cut me to the heart! My father had often told me the story of a friend who had plunged into the river to save me as I was drowning, while an infant, and who, in consequence of a cold then taken, had been made a cripple by inflammatory rheumatism; and this was the man I had made a butt of ridicule, and a laughing-stock for my companions!

"I tell you, boys and girls, I would give a great deal to have the memory of that event taken away. If ever you are tempted as I was, remember that, while no good can come of sport whereby the feelings of others are wounded, you may be laying up for yourselves life-long painful recollections."

